# How healthy participants value additional diagnostic testing with amyloid-PET in patients diagnosed with mild cognitive impairment — a bidding game experiment

**DOI:** 10.1186/s13195-023-01346-y

**Published:** 2023-11-28

**Authors:** I. S. van Maurik, E. D. Bakker, A. A. J. M. van Unnik, H. M. Broulikova, M. D. Zwan, E. van de Giessen, J. Berkhof, F. H. Bouwman, J. E. Bosmans, W. M. van der Flier

**Affiliations:** 1 Alzheimer Center Amsterdam, Neurology, Vrije Universiteit Amsterdam, Amsterdam UMC Location VUmc, De Boelelaan 1118, Amsterdam, The Netherlands; 2 Amsterdam Neuroscience, Neurodegeneration, Amsterdam, The Netherlands; 3 Epidemiology and Data Science, Amsterdam UMC Location Vrije Universiteit Amsterdam, De Boelelaan 1117, Amsterdam, The Netherlands; 4 Amsterdam Public Health, Methodology, Amsterdam, The Netherlands; 5 Department of Health Sciences, Faculty of Science, Vrije Universiteit Amsterdam, Amsterdam Public Health Research Institute, Amsterdam, The Netherlands; 6 Department of Radiology and Nuclear Medicine, Vrije Universiteit Amsterdam, Amsterdam UMC Location VUmc, Amsterdam, the Netherlands

**Keywords:** Alzheimer’s disease, Amyloid-PET, Bidding game

## Abstract

**Background:**

To estimate the perceived value of additional testing with amyloid-PET in Euros in healthy participants acting as analogue patients with mild cognitive impairment (MCI).

**Methods:**

One thousand four hundred thirty-one healthy participants acting as analogue MCI patients (mean age 65 ± 8, 929 (75%) female) were recruited via the Dutch Brain Research Registry. Participants were asked to identify with a presented case (video vignette) of an MCI patient and asked whether they would prefer additional diagnostic testing with amyloid PET in this situation. If yes, respondents were asked how much they would be willing to pay for additional diagnostic testing. Monetary value was elicited via a bidding game in which participants were randomized over three conditions: (A) additional testing results in better patient management, (B) Same as condition A and a delay in institutionalization of 3 months, and (C) same as A and a delay in institutionalization of 6 months. Participants who were not willing to take a test were compared with participants who were willing to take a test using logit models. The highest monetary value per condition was analyzed using random-parameter mixed models.

**Results:**

The vast majority of participants acting as analogue MCI patients (87% (*n* = 1238)) preferred additional testing with amyloid PET. Participants who were not interested were more often female (OR = 1.61 95% CI [1.09–2.40]) and expressed fewer worries to get AD (OR = 0.64 [0.47–0.87]). The median “a priori” (i.e., before randomization) monetary value of additional diagnostic testing was €1500 (IQR 500–1500). If an additional amyloid PET resulted in better patient management (not further specified; condition A), participants were willing to pay a median price of €2000 (IQR = 1000–3500). Participants were willing to pay significantly more than condition A (better patient management) if amyloid-PET testing additionally resulted in a delay in institutionalization of 3 months (€530 [255–805] on top of €2000, condition B) or 6 months (€596 [187–1005] on top of €2000, condition C).

**Conclusions:**

Members of the general population acting as MCI patients are willing to pay a substantial amount of money for amyloid-PET and this increases when diagnostic testing leads to better patient management and the prospect to live longer at home.

**Supplementary Information:**

The online version contains supplementary material available at 10.1186/s13195-023-01346-y.

## Introduction

Many patients who visit a memory clinic are diagnosed with mild cognitive impairment (MCI) [1]. The underlying cause of MCI can be heterogeneous, ranging from neurodegenerative disease, most often Alzheimer’s disease (AD), to more benign causes, such as depression, burn-out, or sleep problems. The development of new diagnostic tools, including amyloid-PET, and biomarkers in cerebrospinal fluid or blood, enables a more accurate etiological diagnosis of Alzheimer’s disease [2–5]. Amyloid PET has the advantage over biomarkers in CSF or blood, in that it quantifies and visualizes amyloid burden, yet is rather expensive.

Making use of amyloid PET, a diagnosis can already be made prior to the stage of dementia, e.g., in the stage of MCI. However, an amyloid-PET scan does not provide full certainty about the underlying cause, and it remains difficult to predict risk and time to onset of dementia precisely [6]. In the absence of curative treatment options, a diagnosis of Alzheimer’s disease has no direct effect on treatment [7].

On the other hand, a diagnosis may be important for patients and their caregivers to understand the nature of a patient’s complaints [1, 8]. Also, an accurate diagnosis is the gateway to proper care. Earlier studies showed that the use of amyloid-PET increases diagnostic certainty and impacts patient management [3–5, 9–11]. Moreover, patients who underwent amyloid-PET during the diagnostic work-up were less often admitted to the hospital and had a lower rate of institutionalization and decreased healthcare costs in the years following diagnosis [12]. This shows that amyloid-PET has the potential to result in health benefits, even in the absence of curative treatment for AD.

Stated preference studies and discrete choice experiments are examples of survey methods that elicit to assign monetary values to the outcome of a health care program or problem under evaluation [13]. Several of such studies have been performed recently, all focusing on situations where a potential treatment is available and show a high willingness to accept risk for potential treatment [14–16]. One study examined the willingness to accept testing for AD, the perceived monetary values of AD test information, and the accuracy of such information [17]. However, to date there is a lack of studies that investigate how (potential) patients value diagnostic information by amyloid-PET in a situation where curative treatment options are not available. We aimed to determine in a sample of healthy participants acting as analogue MCI patients how they value additional diagnostic testing with amyloid-PET, quantified in Euros.

## Methods

### Study design and experimental conditions

We performed a randomized online survey study where healthy participants, acting as analogue MCI patients [18, 19], were randomized over three conditions and an ascending and descending sub-scheme. We elicited an “a priori” monetary value (i.e., before randomization) and an “a posteriori” monetary value (after randomization) for additional diagnostic testing with amyloid-PET. More detailed information is described in Supplemental Text 1 and below (“ Survey”). Upon randomization, the following information was provided for the three conditions: condition A, the result of the amyloid-PET scan results in better patient management (not further specified); condition B, same as condition A and a delay in institutionalization of 3 months; condition C, same as A and a delay in institutionalization of 6 months. Within each condition, half of the participants were shown questions on costs for diagnostics in an ascending and half in a descending order.

The study was reviewed by the board of the Medical Ethics Committee of Amsterdam University Medical Center, location VU Medical Center. All participants gave informed consent.

### Participants

Healthy individuals who registered for the Dutch Brain Health Registry and with an age over 50 years old were invited to participate in this study [20]. *N* = 5228 eligible people received a study invitation between August 2021 and February 2022. Those who demonstrated interest in participation (*n* = 1645) received a personal link to the online questionnaire. Of those interested, *n* = 1526 started the online questionnaire and complete data of *n* = 1453 participants were available for analysis.

### Survey

Participants were asked to identify as best as possible with the presented case of a patient with MCI and his daughter who were visiting a memory clinic. The full text of the script and information provided to participants acting as analogue MCI patients can be found in the supplement. The case was presented in a short video made with professional actors and shows the patient in the waiting room overthinking his cognitive complaints [21]. After this video, participants were provided with more written information on MCI and the arguments against (there is no treatment, difficult to predict whether a patient will get dementia) and in favor of (understanding the nature of cognitive complaints and could help with long-term (care) planning) additional diagnostic testing. Participants were then asked whether, in the described situation, they would prefer to undergo additional diagnostic testing using an amyloid-PET scan. Participants who answered “yes” were subsequently asked how much this amyloid-PET scan may cost when paid for by the insurer as is standard in the Dutch health care system: €0, €500, €1500, €2500, €3000. The answer options were based on what would be a realistic price for amyloid-PET in a clinical setting. Such answer options and the order of how these answer options are shown may influence how participants value additional testing. Therefore, we randomized participants to receive the answer options in descending or ascending order. The chosen answer was the starting point for the bidding game questions.

For the bidding game, participants acting as analogue MCI patients were randomized over three conditions A, B, and C (see also Table 1) and were asked how much they would be willing to pay for amyloid-PET in a bidding game design; if answering “yes,” the next question is €500 higher, if answering “no” the next question is €500 lower, eventually eliciting the highest monetary value for amyloid-PET.

**Table 1:** Sample characteristics

	**Not interested in testing**	**Interested in testing**			
		**All**	**Condition A**	**Condition B**	**Condition C**
	***N* = 193 (13%)**	***N* = 1238 (87%)**	***N* = 408**	***N* = 413**	***N* = 417**
**Age**	66 ± 7	65 ± 8	65 ± 8	64 ± 8	66 ± 8
**Female sex, *n* (%)**	160 (83%)	929 (75%)	307 (75%)	314 (76%)	308 (74%)
**Education, Verhage ** [22]	6 ± 1	6 ± 1	6 ± 1	6 ± 1	6 ± 1
**Positive family history**	140 (73%)	905 (73%)	312 (76%)	296 (72%)	297 (71%)
**Cared for person with dementia, *n* (%)**	72 (51%)	444 (49%)	156 (50%)	136 (46%)	152 (51%)
**Income, *n* (%)**					
< €1000	3 (2%)	18 (2%)	5 (1%)	3 (1%)	10 (2%)
€1000–€2500	70 (36%)	367 (30%)	121 (30%)	117 (28%)	129 (31%)
€2500–€5000	83 (43%)	537 (44%)	171 (42%)	182 (44%)	184 (44%)
> €5000	14 (7%)	141 (11%)	44 (11%)	56 (14%)	41 (10%)
Unknown	22 (11%)	172 (14%)	66 (16%)	53 (13%)	53 (13%)
**Attended memory clinic, *n* (%)**					
Yes, for myself	6 (3%)	46 (4%)	18 (4%)	14 (3%)	14 (3%)
Yes, for my spouse	28 (15%)	190 (15%)	69 (17%)	65 (16%)	56 (13%)
Yes, for myself and my spouse	2 (1%)	21 (2%)	6 (1%)	7 (2%)	8 (2%)
Not sure	8 (4%)	47 (4%)	16 (4%)	15 (4%)	16 (4%)
No	149 (77%)	934 (75%)	299 (73%)	312 (76%)	323 (77%)
**Worries for AD**	103 (54%)	797 (64%)	264 (65%)	271 (66%)	263 (63%)
**EQ-5D-5L utility score**	0.87 ± 0.13	0.86 ± 0.15	0.86 ± 0.15	0.87 ± 0.15	0.85 ± 0.17
**EQ-5D VAS**	84 ± 19	84 ± 17	84 ± 18	84 ± 17	83 ± 17
**STAI**	13 ± 2	13 ± 2	13 ± 3	13 ± 2	13 ± 2
**Device used to complete questionnaire**					
Desktop	111 (58%)	741 (60%)	240 (59%)	242 (59%)	259 (62%)
Tablet	24 (12%)	96 (8%)	33 (8%)	34 (8%)	29 (7%)
Smartphone	193 (30%)	401 (32%)	135 (33%)	137 (33%)	129 (31%)

*AD* Alzheimer’s disease, *EQ-5D-5L* 5-level version of EuroQol-5 Dimension, *VAS* visual analog scale, *STAI* State-Trait Anxiety Inventory. Condition A, the result of the amyloid-PET scan results in better patient management (not further specified); condition B, same as condition A and a delay in institutionalization of 3 months; condition C, same as A and a delay in institutionalization of 6 months

The survey also collected information on family history, caregiver status (caring or have cared for someone with dementia), income, education level, quality of life (measured with the 5-level version of EuroQol-5 Dimension (EQ-5D-5L)) and current anxiety level (short-form State-Trait Anxiety Inventory (S-STAI-S). The survey was developed with input from existing literature, health care professionals, and health economic evaluation professionals.

### Data analysis

First, we analyzed the respondents’ “a priori” monetary value for diagnostic testing in patients with MCI. This “a priori” estimate is based on their stated preference with regard to diagnostic testing and the subsequent monetary value after the video in which the MCI case was presented, but before randomization on conditions A, B, and C varying in health benefit. To identify characteristics related to the willingness to undergo additional testing in case of MCI, characteristics of respondents (demographics, caregivers status, experiences, and own health) willing to undergo diagnostic testing were compared with respondents who were not willing to undergo diagnostic testing using logit models.

Second, we analyzed the maximum price for amyloid-PET, elicited via contingent valuation. To compare the difference in how much people were willing to pay for condition A with that for conditions B and C a mixed random-parameter model was used. The “a priori” willingness to pay was included as a random effect and the model was adjusted for the order in which the “a priori” answer options were shown (ascending or descending). Confidence intervals were estimated using bootstrapping with 5000 replications. In a sub-analysis, we reran the model on the highest monetary value by applying 95 percentile winsorization on extreme values identified as outliers that are 300% of the IQR (i.e., above the median).

## Results

### Participant characteristics

Overall participants acting as analogue MCI patients were 65 ± 8 years old and predominantly female (*n* = 1089 (76%)). The majority (*n* = 1045 (73%)) reported to have a family member or friend with dementia, of whom 514 (49%) provided informal care. A total of 193 (13%) participants chose the no-test option and did not express interest in diagnostic testing in case of an MCI diagnosis. The remaining *n* = 1238 (87%) were randomized over three conditions, that differed in the health benefit that additional testing by amyloid PET would incur. Characteristics of participants are summarized in Table 1. We found no significant differences in sample characteristics between conditions.

### Characterizing those not interested in additional testing

Univariable models showed that participants who were not interested in diagnostic testing were more often female (OR = 1.61 95% CI [1.09–2.40]) and less often worried about getting AD in the future (OR = 0.64 95% CI [0.47–0.87]) than participants who showed interest in diagnostic testing. No other differences were found.

### A priori monetary value of additional testing

The *n* = 1238 (87%) participants who demonstrated interest in diagnostic testing based on the video vignette, attributed a median a priori monetary value of €1500 (IQR 500–1500) on additional testing using amyloid PET. Most participants (*N* = 1127; 91%) were willing to pay €500 or more, while a minority of *n* = 112 (9%) chose a value of additional testing of €0. Univariable analyses showed that these participants were less often worried for future AD (OR = 0.63 95% CI [0.43–0.93], lower educated (OR = 0.71 95% CI [0.57–0.88]), and of higher age (OR = 1.03 95% CI [1.00–1.05]) than participants who were willing to pay €500 or more. Survey completion time was not associated with the a priori monetary value of additional testing.

### The effect of potential delayed institutionalization on the highest monetary value

After randomization, participants acting as analogue MCI patients were asked their (maximum) monetary value for additional diagnostic testing. Median a posteriori monetary value per condition are shown in Supplemental Table 1. In all conditions, participants indicated a higher a posteriori price than selected a priori. Participants were willing to pay significantly more than condition A (better patient management) if amyloid-PET testing resulted additionally in a delay in institutionalization of 3 months (€530 on top of €2000 95% CI [255–05], condition B or 6 months (€596 on top of €2000 95% CI [187–1005], condition C)). The difference in additional costs between condition B and condition C was not significant (€66 [− 437–304]). Adding completion time of the survey to the model did not confound the results. Winsorization of extreme values did not change results (Supplemental Table 2).

## Discussion

We show that the majority of people acting as analogue MCI patients are willing to undergo additional testing with amyloid PET in order to receive more specific information on the cause of their cognitive decline. Even in the absence of curative treatment options, people value such information. If an additional amyloid PET resulted in better patient management alone, participants were willing to pay a price of €2000. If additional amyloid-PET testing also resulted in a delay in institutionalization, participants were willing to pay roughly €500 more.

In the current study, we asked participants to identify with a patient who just received a MCI diagnosis. Based on the case we presented, most participants preferred additional testing to learn more about the cause of MCI. In discrete choice experiments and contingent valuations, income and a participant’s socio-economic background may influence whether someone is likely to pay a (high) amount of money for a service or intervention [23]. Although we found differences in the willingness to undergo additional testing between men and women, and between people who worry more or less about future dementia, the preference for additional testing was not associated with income, education level, or participants’ quality of life. While we can reason why someone who is not worried about future dementia is less likely to prefer (expensive) additional diagnostic testing, the sex difference seems less obvious. However, this is in line with a previous study that showed that women are willing to pay less for goods with uncertain revenues [24].

From earlier studies in a clinical setting, we know that amyloid-PET contributes to higher clinician confidence in the diagnosis and better patient management [9, 25]. In addition, using amyloid PET to obtain a more accurate diagnosis resulted in lower healthcare costs and a lower rate of institutionalization [12]. In particular, we observed a delay in institutionalization of more than one year, which is larger than we described in the scenarios in the current study. While it is unlikely that such a delay would be attributable to the amyloid PET scan per se, we reason that the PET scan may contribute to a more accurate diagnosis, which in turn leads to better fitting care, and less crises and hospitalizations further in the disease trajectory. In line with this reasoning, MCI diagnostic guidelines increasingly acknowledge the value of an accurate and accurately communicated diagnosis at the level of the patient and care partner, emphasizing the personal value of accurate information [1, 8, 21]. The current study adds to this previous research by showing how much healthy participants are willing to pay for better patient management and delay in institutionalization as a result of additional diagnostic testing.

The monetary value strongly depends on how detailed the information on costs in the questionnaire is. Where in an earlier study the monetary value for AD testing, either with cerebrospinal fluid or imaging, has been estimated at €700 the estimates of the current study are substantially higher [17]. However, the former study did not provide participants with information on what such a test could potentially cost. We think it is important to provide participants with information on what a realistic price of such diagnostic interventions could be, so that they can make an informed decision. However, cost estimates may differ from country to country and the relatively high cost estimate from the current study may very well be influenced by the fact that all participants were Dutch citizens with health insurance. In the Netherlands, out-of-pocket costs vary according to the chosen reimbursement package, between a minimum of €385 and a maximum of €885 per year. In other countries, the healthcare systems are arranged differently, as are the personal costs for healthcare. In the Netherlands, people might think that certain health care services are free, because a very large share is paid by the health insurer. A consequence of this may be that they report a higher monetary value than in other countries with different healthcare systems. Therefore, the results of this study have limited generalizability. Still, we show that people value additional information with regard to the underlying pathology of their complaints, and even more so if this information results in health benefits.

In our study, we focused on amyloid PET, rather than biomarkers in CSF or blood. Amyloid PET has some specific characteristics — i.e., quantifies and visualizes amyloid burden and comes at rather high monetary costs — that make it of interest for our study. Similar studies could be done for CSF or blood-based biomarkers, and in the future, there could also be designs asking participants to weigh pros and cons of the different modalities. In this first step, however, we chose for a straightforward design focusing on only one test modality, to keep the number of choices participants were faced with limited. We feel that rather as exact monetary value of a specific test (i.e., amyloid PET scan), the results should be interpreted in a more generalized way; people are willing to pay for an etiological diagnosis, even in the absence of curative treatment. With market access of the first generation of disease-modifying treatments, interest in etiological diagnosis may increase even further. To keep healthcare accessible and scalable, it will be of the utmost importance to make use of blood-based biomarkers, whose swift development allows future role out in clinical practice.

The AD field is moving fast and with the results of lecanemab and donanemab, the landscape is about to change. In Europe, these drugs are being assessed but there will be no advice from the European Medicines Agency before 2025, which is the target group of the current study. This means that in the near future, patients will not (yet) have access to these therapies. Demonstrating the health benefits of diagnostic tests when curative treatment options are not available is challenging. In case of Alzheimer’s disease, such health benefits are likely to take place later in the disease process, potentially years after a diagnosis has been made. This is further complicated by the fact that potential cost savings will most likely be observed in the social care setting, for example by a delay in institutionalization, while the costs of diagnosis are payed in the healthcare setting. This shift of costs between settings is a major challenge for the healthcare sector, since this sector needs to bear the costs but will not gain from the benefits [26]. In the current study, we clearly show that in scenarios where the health benefit is larger, people are willing to pay more for a diagnostic test. Moreover, even without specified health benefit, participants were already willing to pay substantially for the test.

Among the strengths of the current study is that we recruited cognitively healthy participants via the Dutch Brain Research Registery [20] and the use of a professionally designed case vignette to introduce the case of MCI [21]. By doing so, we believe that the participants of the current study could realistically identify with the situation in which someone is diagnosed with MCI. Participants in this study were particularly highly educated. Although this might have been an advantage, as contingent valuation questions are complex, it limits the generalizability of the findings of this study. Family income of participants was relatively high, which might have inflated the price values that we have found. When presenting the answer options for costs for amyloid-PET, patients were randomized into an ascending and a descending sub-scheme in order to cancel out the methodological issue of ordering effects in bidding game experiments [27]. There might have been some suggestiveness in the arguments presented, and the arguments in favor and against amyloid-PET scans were not randomized, and therefore, we cannot rule out primacy or recency effects. Given the complexity of the information and the task at hand (i.e., remember information and act as analogue patients), a primacy effect might have been more pronounced resulting. Since arguments against amyloid-PET were always given first, people might have been more inclined to answer “no” to additional testing. Nonetheless, the vast majority of participants indicated to be interested in additional testing.

## Conclusion

In conclusion, we showed that the preference and related monetary value for additional amyloid-PET testing in MCI patients is high despite the absence of curative treatment and increases when diagnostic testing leads to better patients management and the ability to live longer at home.

### Supplementary Information

**Additional file 1: Supplemental Information 1.** Information provided to participants in the survey. **Supplemental Table 1.** Highest monetary values by health benefit. **Supplemental Table 2.** Willingness-to-pay results from random effect model after winsorization.

## Data Availability

The datasets analyzed during the current study are available from the corresponding author on reasonable request.

## Abbreviations

AD: Alzheimer’s disease
EQ-5D-DL: 5-Level version of EuroQol-5 Dimension
IQR: Interquartile range
MCI: Mild cognitive impairment
OR: Odds ratio
PET: Positron emission tomography
STAI: State-Trait Anxiety Inventory
95% CI: 95% Confidence interval
