# Supplementary material for: How healthy participants value additional diagnostic testing with amyloid-PET in patients diagnosed with mild cognitive impairment — a bidding game experiment
Source: Alzheimers Res Ther. 2023 Nov 28;15:208. doi: 10.1186/s13195-023-01346-y (PMC10683285; doi:10.1186/s13195-023-01346-y)
Supplement: Supplementary file 1 — Additional file 1: Supplemental Information 1. Information provided to participants in the survey. Supplemental Table 1. Highest monetary values by health benefit. Supplemental Table 2. Willingness-to-pay results from random effect model after winsorization. [file 13195_2023_1346_MOESM1_ESM.docx]

**Supplemental Material**

**How people value additional diagnostic testing with amyloid-PET when diagnosed with MCI**

I.S. van Maurik, E.D. Bakker, A. van Unnik, H. Broulikova, M.D. Zwan, N.C. Visser, E. van de Giessen, J. Berkhof, F. Bouwman, J. Bosmans, W.M. van der Flier

**Supplemental information 1. Information provided to participants in the survey**

**Supplemental Table 1. Highest monetary values by health benefit**

**Supplemental table 2. Willingness-to-pay results from random effect model after winsorization**

**Supplemental information 1. Information provided to participants in the survey**

Alzheimer's develops gradually over many years. Previously, you could only make a definite diagnosis of Alzheimer's after death, when abnormal accumulations of Alzheimer's proteins in brain tissue were demonstrated via autopsy. For about 10 years, the Alzheimer's PET scan has made it possible to image Alzheimer's brain damage during life.

When a diagnosis of dementia is made, the disease has already been active in the brain for years. With help of the Alzheimer's PET scan, the disease can be detected even before the onset of dementia. For example, in the stage of mild cognitive impairment (MCI).

Start video with the text:

*“I am Cornelis Janssen. I've had some memory problems and difficulty concentrating for some time now. Of course I was a bit worried about that, but it's also very busy at work. I actually assumed that was why. Until my birthday. I went home a little earlier, because we were going to have a big dinner with the family. Then suddenly I got a call from work that my appointment was there. Where was I? I really couldn't remember making that appointment and so I hadn't put it in my agenda either. While it was an important customer!*

*My wife and daughter were also worried for a while, but this shocked them a bit. They thought I should go to the doctor.*

*Well, then I ended up here a month ago, in the memory clinic. Last time they did all kinds of tests, with all kinds of assignments and word lists that I had to memorize. A week later I got the results.*

*The doctor talked about disorders. That's what they found with those tests. Disorder of memory and learning ability. So.. I sometimes forget an appointment, but who doesn't? Isn't that part of getting older? I didn't really expect a disorder.”*

When Mild Cognitive Disorders (MCI) have been diagnosed, it is not always investigated whether this is due to Alzheimer's Disease, for example with the aid of an Alzheimer's PET scan. Demonstrating Alzheimer's brain damage gives a clearly increased risk of dementia, but no certainty. It is also difficult to predict exactly when dementia will occur. As long as there are no drugs, a pre-dementia diagnosis of Alzheimer's has no direct effect on treatment, and this may be a reason not to do the diagnostic test. On the other hand, a diagnosis is important for patients to understand the cause of their complaints, to discuss the prognosis and to make plans for the long term.

In short: some patients and their relatives would like to know the cause of their complaints, even if there is no cure to stop the disease. Other patients and their relatives prefer to wait and let the future tell what happens. In addition, an Alzheimer's PET scan is expensive.

Can the absence of medicines that stop the disease be a reason not to provide information about the disease? In other words: what is information about the cause of your complaints worth to you in money, or: what can the Alzheimer's PET scan cost? It is important that you bear in mind that the costs for the Alzheimer's PET scan are paid by the health insurance, of course after deduction of your deductible.

Question 1: Imagine going to the memory clinic. Just like Cornelis Janssen in the video, you have mild cognitive impairment. Would you like to get an Alzheimer's PET scan?

c Yes

c No [order of answer options varied]

Question 2. How much can an Alzheimer's PET scan cost?

c €0

c €500

c €1500

c €2500

c €3000 [order of answer options varied]

**Supplemental Table 1. Highest monetary values by health benefit**

|  | **Median** | **IQR** |
| --- | --- | --- |
| **Condition A : better patient management** | €2000 | 1000-3500 |
| **Condition B : A + 3 months delay in institutionalization** | €2500 | 1000-4000 |
| **Condition C : A + 6 months delay in institutionalization** | €2000 | 1000-4000 |

**Supplemental table 2. Willingness-to-pay results from random effect model after winsorization**

|  | **β** | **Bootstrapped 95%CI** |
| --- | --- | --- |
| **Intercept** | 2827 | 2656 -2998 |
| **Condition A : better patient management** | reference |  |
| **Condition B : A + 3 months delay in institutionalization** | €463 | 246-680 |
| **Condition C : A + 6 months delay in institutionalization** | €289 | 60-517 |
